# Post-Ejection Reorientation of Rice Pot Seedlings for Automated Transplanting: Mechanism and Optimization of a Cam-Driven Continuous Picking System

**DOI:** 10.3390/plants15203078

**Published:** 2026-10-09

**Authors:** Jinfeng Wu, Can Li, Huolong Huang, Peng Xu, Peng Fang, Jiajia Yu, Jinping Cai, Xiongfei Chen, Muhua Liu

**Affiliations:** 1 College of Engineering, Jiangxi Agricultural University, Nanchang 330045, China; wujinfeng@stu.jxau.edu.cn (J.W.); canli@jxau.edu.cn (C.L.); huanghuol@webmail.hzau.edu.cn (H.H.); xupeng@jxau.edu.cn (P.X.); fangpeng@jxau.edu.cn (P.F.); yujiajia316@jxau.edu.cn (J.Y.); caijinping197@jxau.edu.cn (J.C.); 2 Jiangxi Provincial Key Laboratory of Modern Agricultural Equipment, Nanchang 330045, China

**Keywords:** precision agriculture, automated rice transplanting, cam-driven continuous picking system, rice pot seedling, post-ejection reorientation

## Abstract

Precision rice transplanting requires pot seedlings to be released intact and in a repeatable orientation for coordinated conveying and planting. This study investigated post-ejection reorientation in a cam-driven continuous picking system and proposed a root-ball-leading, shoot-trailing target orientation. Based on system kinematics, seedling mass distribution, and contact conditions, the process was divided into four stages: initial ejection, ejection-induced sliding, in-cell rotation, and post-release rotation. High-speed photography resolved stages 2–4, and the observed sequence was qualitatively consistent with the theoretical analysis. A Box–Behnken design evaluated the effects of root-ball moisture content, seedling age, and picking frequency on reorientation success rate and height. Within the tested ranges, multiresponse optimization identified a practical root-ball moisture target of 53% (maintained within ±2 percentage points), the 2.5-leaf stage, and 4 s^−1^ as the recommended combination. At the corresponding numerical optimum, the models predicted a reorientation success rate of 93.75% and a reorientation height of 35.08 cm, while bench confirmation at the practical setting yielded 93.33% and 33.70 cm, respectively. By focusing on post-ejection orientation under controlled bench conditions, this study provides a mechanistic and design basis for integrating continuous picking with downstream conveying and planting units in precision automated transplanting.

## 1. Introduction

Precision agricultural mechanization increasingly relies on automated field operations that translate variations in crop and substrate conditions into controllable machine settings. In rice production, pot-seedling transplanting promotes rapid regreening, maintains low tillering-node positions, supports strong canopy recovery, and improves field efficiency and post-transplant growth [1,2,3]. Consistently realizing these agronomic and productivity-related benefits requires coordinated and reliable mechanized operations. Mechanized transplanting systems integrate front-end seedling removal with downstream conveying and planting [4,5]. Among these operations, seedling picking is particularly important because the release state generated by the picking unit establishes the initial conditions for all subsequent motion. Therefore, repeatable post-ejection orientation is essential for reliable coordination between seedling picking and downstream handling in automated transplanting.

Development of automated rice pot-seedling transplanting began relatively early in Japan. Iseki’s PZP80 uses planting arms and requires precise coordination among mechanisms, whereas Minoru’s RXG80D-GS integrates automatic tray feeding and empty-tray collection but requires dedicated trays and strict synchronization. Related systems have also been developed in China since the 1980s. However, tray compatibility, coordination among operations, and supporting technologies remain limited, and most systems are still at the experimental or demonstration stage [6,7,8].

Research on automated cell-tray seedling handling has focused mainly on picking mechanisms and interactions among end effectors, seedlings, and root balls [9,10,11]. For plug seedlings, end-effector design and motion control have been investigated to support automatic transplanting [9,11]. Rice seedling-picking systems generally use pneumatic, ejection-type, or gripping-and-pulling mechanisms. Wang et al. used air jets in a pneumatic orderly throwing system [12]; Yuan et al. combined a carrier, advancing mechanism, and guide device for ejection-based release [3]; and Cai et al. used cam-controlled clamps to vary planting row spacing [13]. Although these approaches improve handling continuity, they mainly evaluate successful tray removal, root-ball integrity, and mechanism performance. The relationship between the release state generated by the picking mechanism and subsequent seedling orientation remains insufficiently characterized.

Seedling motion and orientation during automated conveying and planting affect operational quality and post-transplant establishment [14]. Zhang et al. combined theoretical modeling with high-speed photography to study seedling motion during aerial fall and passage through a guide tube [15]; Ma et al. simulated cell-tray seedling motion in a separating chute [16]; and Tian et al. developed a Runge–Kutta trajectory model whose estimates closely matched high-speed observations [17]. However, these studies largely prescribed initial motion conditions or examined downstream trajectories; how the picking system forms the release orientation has received comparatively little attention. This upstream uncertainty limits precise matching between machine actuation, seedling state, and downstream planting requirements.

Continuous automated transplanting requires seedlings not only to leave tray cells intact but also to attain an orientation compatible with downstream conveying and planting, within the limited drop space of each picking cycle. Picking frequency, seedling condition, and contact with the seedling-separating plate jointly determine the release state. Variations can shift landing positions or cause collisions; therefore, picking success rate alone does not adequately describe compatibility among automated handling modules [18]. For precision agricultural machinery, reorientation success rate and reorientation height provide complementary process indicators that translate orientation consistency and required transfer space into controllable design targets.

The root-ball-leading, shoot-trailing orientation was selected as an intermediate transfer posture rather than the final field posture. Mechanically, the compact root ball provides a stable leading end, while the flexible shoot trails behind; this configuration is expected to reduce the risks of shoot-first entry, outlet entanglement, and uncontrolled rotation. Previous studies have shown that seedling orientation and wall contact affect motion through guide tubes and separating chutes [15,16], while Cai et al. [13] reported that controlling the delivery posture reduced the average inverted-seedling rate to 3.45%. Moreover, the initial angle affects the final angle, landing distance, and terminal velocity [17], whereas falling posture influences seedling–planter collisions, root-ball damage, and planting uprightness [18]. Thus, after downward redirection, this orientation facilitates root-ball-first entry and provides a favorable initial condition for orderly conveying and upright establishment [14].

Previous studies have generally focused either on tray removal and root-ball integrity or on downstream motion under prescribed initial conditions, with limited attention to how the release state forms during picking. In contrast, the present study treats release orientation as an outcome of the coupled ejector-pin–root-ball–tray–separating-plate interaction, and tracks its evolution from ejection to the target orientation. Its central contribution is therefore an upstream process model linking continuous picking to the initial conditions required for downstream automated handling, rather than another free-fall trajectory model.

To address this gap, the present study employed a laboratory prototype of a cam-driven continuous picking system developed in 2025 by our research team at Jiangxi Agricultural University for the continuous ejection of rice pot seedlings from cell trays [19]. The system has not yet been incorporated into a commercial rice pot-seedling transplanter. The objectives were to (1) establish a four-stage process model for the formation of the root-ball-leading, shoot-trailing orientation based on system kinematics, seedling properties, and contact conditions; (2) use high-speed photography to characterize the observable post-ejection reorientation stages; and (3) optimize the combined effects of root-ball moisture content, seedling age, and picking frequency, using reorientation success rate and reorientation height as responses. The results were intended to define controllable machine–seedling conditions for compact integration of continuous picking with downstream automated transplanting and thereby support precision automation of a rice establishment method with documented productivity potential. Agronomic performance and grain yield, however, were outside the scope of the present bench study.

## 2. Materials and Methods

### 2.1. Overall Structure and Operating Principle of the Cam-Driven Continuous Picking System

#### 2.1.1. Overall Structure of the Cam-Driven Continuous Picking System

**Figure 1:**
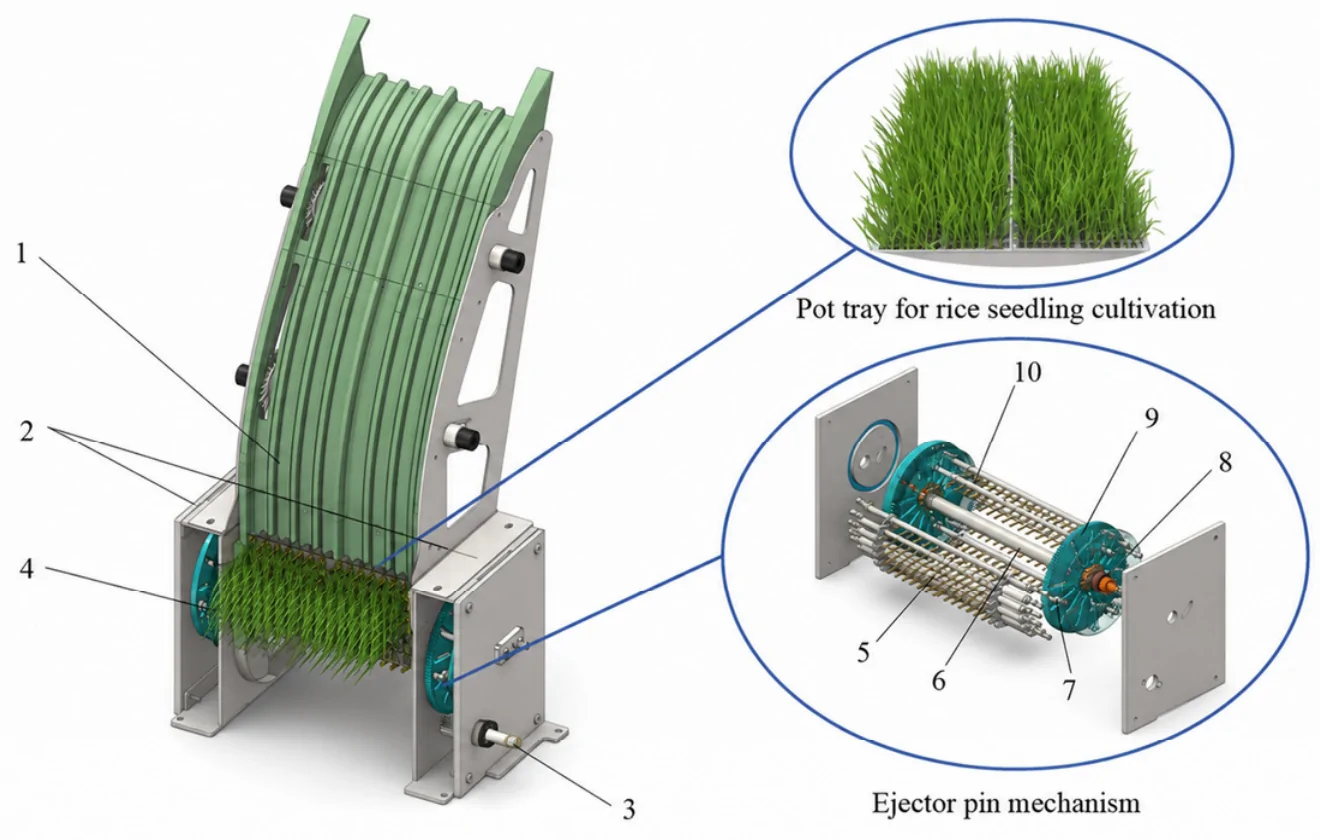
Overall structure of the seedling-picking device. 1. Seedling-tray feeding mechanism; 2. seedling-picking cam housing; 3. drive shaft; 4. ejector-pin mechanism; 5. seedling-picking ejector-pin shaft; 6. main shaft; 7. connecting crankshaft; 8. supporting crankshaft; 9. driving toothed disk; 10. driven disk.

The system comprised a seedling-tray feeding mechanism, two seedling-picking cam housings, a drive shaft, an ejector-pin mechanism, a seedling-tray guide mechanism, a seedling-separating plate, and a baseplate (Figure 1). The two cam housings were mounted symmetrically on the baseplate. Each housing contained a cam groove in its outer sidewall, and the drive shaft rotated the ejector-pin mechanism between the two housings. The feeding and guide mechanisms, together with the seedling-separating plate, conveyed the tray downward while constraining its displacement and bending.

The ejector-pin mechanism comprised 12 pin sets, a mounting shaft, a main shaft, supporting and connecting crankshafts, and driving and driven disks. Each mounting shaft engaged a guide slot in a disk and a cam groove. Together with the supporting and connecting crankshafts, these components formed a parallelogram four-bar linkage, causing each ejector pin to follow the cam groove while reciprocating in its guide slot during rotation.

#### 2.1.2. Operating Principle of the Cam-Driven Continuous Picking System

**Figure 2:**
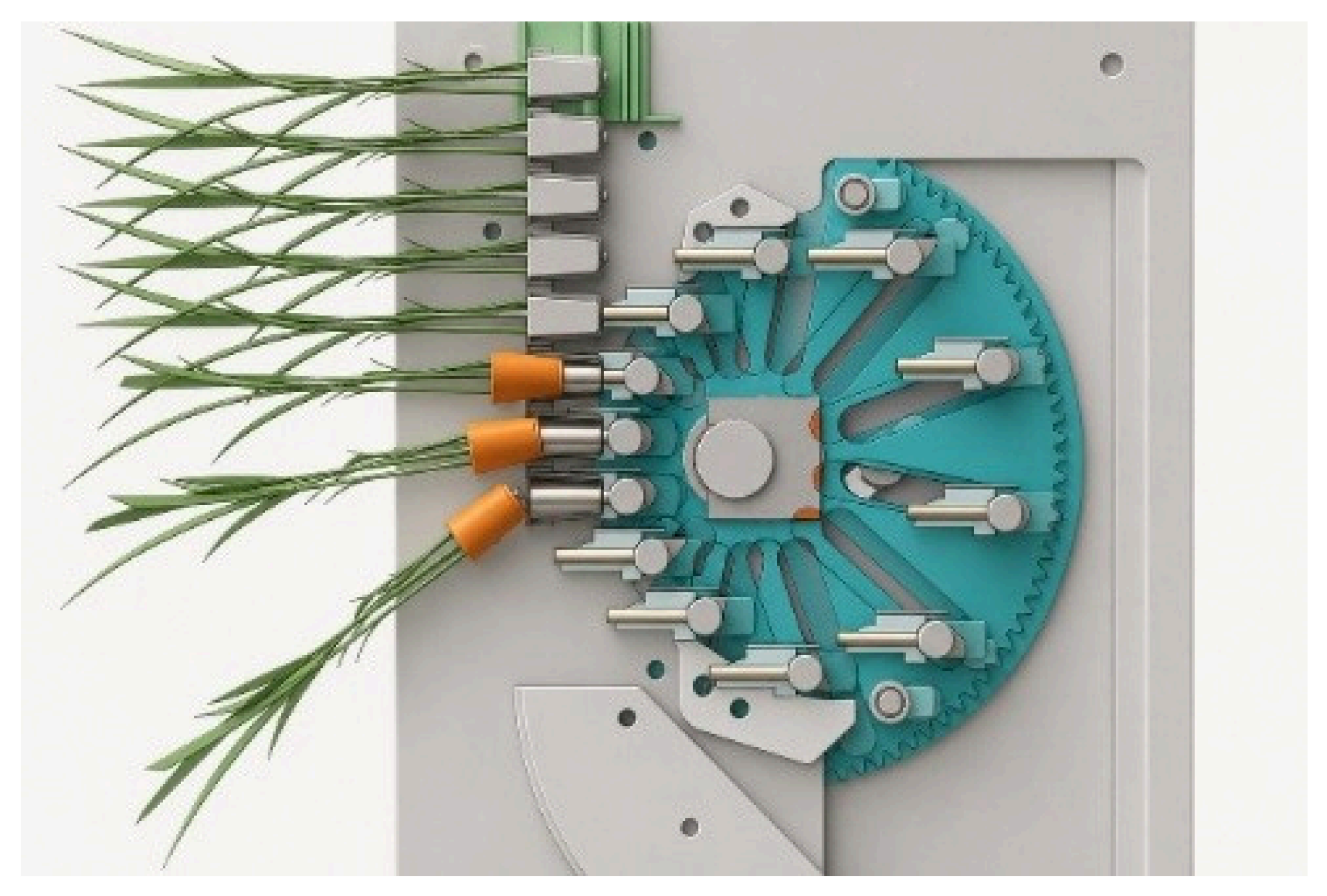
Schematic of the seedling-picking operation.

As shown in Figure 2, the drive shaft continuously rotated the ejector-pin mechanism about the main shaft, while the cam grooves drove radial reciprocation of the pin sets. After the feeding and guide mechanisms positioned the tray, the pins entered the corresponding tray cells in sequence and ejected the seedlings. The cam profile previously developed by Chen et al. coordinated the ejection and return strokes and was defined in polar coordinates as follows [19].

(1) $$\rho =\left\{\begin{array}{ll} \frac{114\varphi }{\pi sin\;\varphi }, & 0\leq \varphi \leq \frac{5\pi }{9}\\ \frac{35-\sqrt{1111.22{tan}^{2}\varphi +3025}}{cos\;\varphi +0.62sin\;\varphi tan\;\varphi }, & \frac{5\pi }{9}<\varphi <\frac{13\pi }{9}\\ \frac{114\left(\varphi -2\pi \right)}{\pi sin\;\varphi }, & \frac{13\pi }{9}\leq \varphi \leq 2\pi \end{array}\right.$$

where ρ is the distance from the origin to the ejector pin (mm); φ is the angle between the X–axis and the line joining the origin to the ejector pin (°).

### 2.2. Motion Analysis of Rice Pot Seedlings During Ejection

#### 2.2.1. Development of the Rice Pot-Seedling Model

Physical properties were measured for Zhongjiazao 17 rice (*Oryza sativa* L.) at seedling ages of 2.5, 3.0, and 3.5 leaves. Seedling height was measured in five randomly selected seedlings at each age, giving mean values of 13, 15, and 17 cm, respectively. For each combination of seedling age and root-ball moisture content, 30 additional seedlings were sampled from three independent seedling trays, with 10 seedlings randomly selected from each tray. These seedlings were used to determine fresh mass and the center-of-mass position relative to the root-ball base, with the latter measured using the suspension method (Table 1). The effects of root-ball moisture content, seedling age, and their interaction were evaluated using two-way analysis of variance, followed by Tukey’s multiple-comparison test at $\left(p<0.05\right)$. Across all treatments, the center of mass ranged from 15.15 to 18.00 mm above the root-ball base and remained within the 24.5 mm-high root ball.

**Figure 3:**
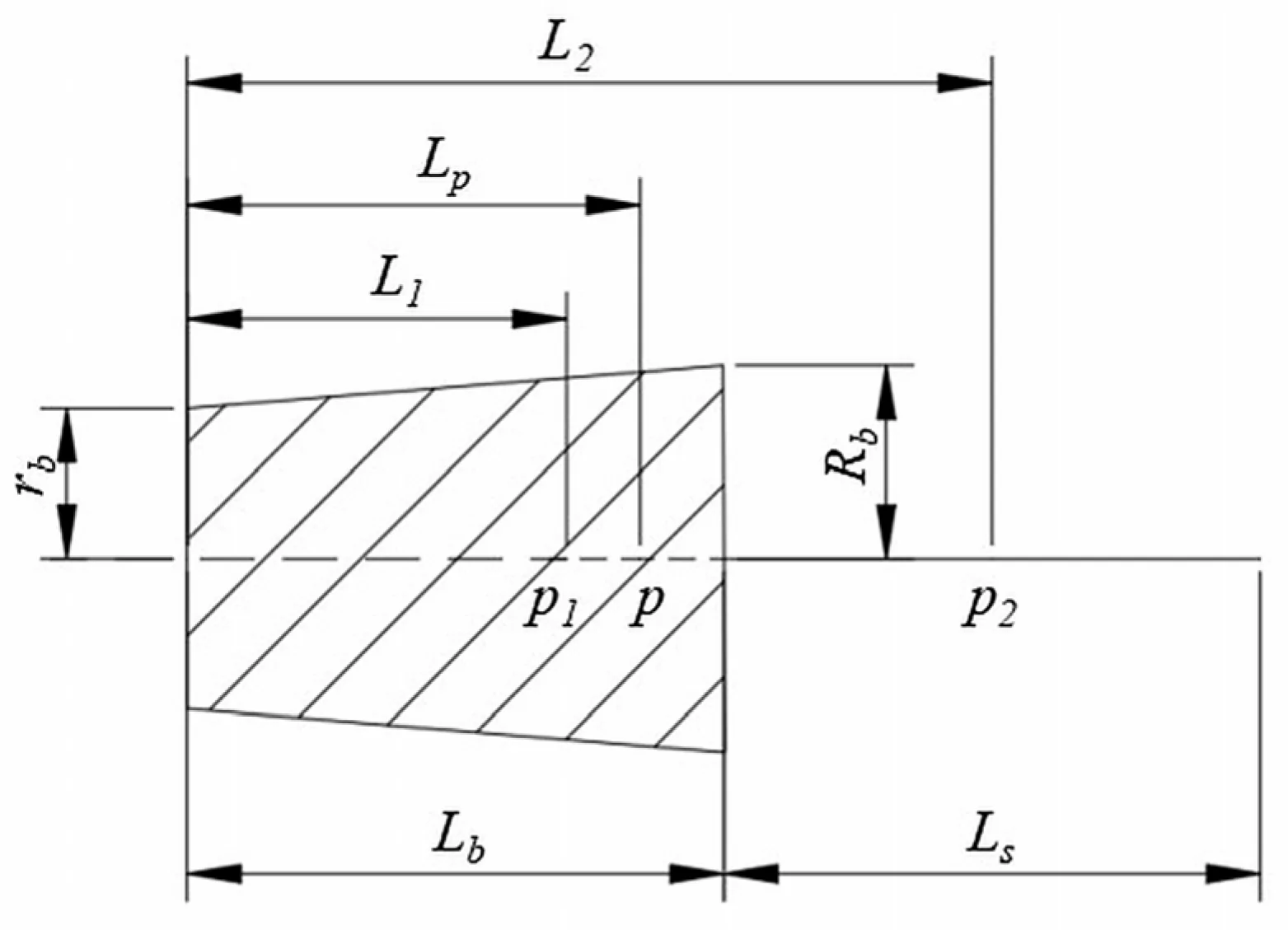
Simplified theoretical model of a rice pot seedling.

A simplified model was constructed to represent seedling mass distribution during ejection. Because the measured center of mass remained within the root-ball portion at all seedling ages, the root ball and shoot were idealized as a uniform conical frustum and a uniform cylindrical rod, respectively, with different densities. The frustum had radii $r_{b}$ and $R_{b}$, length $L_{b}$, and mass $m_{1}$; the rod had length $L_{s}$ and mass $m_{2}$ (Figure 3).

The distance from the conical root-ball base to its center of mass $p_{1}$ is given by:

(2) $$L_{1}=\frac{L_{b}}{4}\left(\frac{R_{b}^{2}+2R_{b}r_{b}+3r_{b}^{2}}{R_{b}^{2}+r_{b}^{2}+R_{b}r_{b}}\right)$$

The distance from the conical root-ball base to the center of mass $p_{2}$ of the straight cylindrical rod is:

(3) $$L_{2}=L_{b}+\frac{L_{s}}{2}$$

Combining Equations (2) and (3) gives the distance from the conical root-ball base to the overall center of mass, *p*, as:

(4) $$L_{p}=\frac{m_{1}L_{b}\left(R_{b}^{2}+2R_{b}r_{b}+3r_{b}^{2}\right)+2m_{2}\left(2L_{b}+L_{s}\right)\left(R_{b}^{2}+r_{b}^{2}+R_{b}r_{b}\right)}{4\left(m_{1}+m_{2}\right)\left(R_{b}^{2}+r_{b}^{2}+R_{b}r_{b}\right)}$$

#### 2.2.2. Ejection and Rotational Motion of Rice Pot Seedlings

In the theoretical derivation, the seedling was modeled as a rigid body undergoing planar motion with a fixed mass distribution; deformation, internal relative motion, and out-of-plane rotation were neglected. Contact at the tray edge was idealized as point contact with short-duration friction. Position V was defined as the loss of root-ball contact with the tray cell; after this point, frictional resistance from brief shoot–plate contact could persist until the shoot separated from the plate. The total vertical aerodynamic drag and transient shoot–plate resistance were approximated by equivalent mean resultants over the short V–VII interval. Angular acceleration was assumed constant, and the vertical velocity at release was neglected. The resulting expressions were therefore interpreted only as theoretical estimates and reference values and were not treated as quantitatively validated descriptions of the actual trajectory.

(1) First stage: Initial ejection

**Figure 4:**
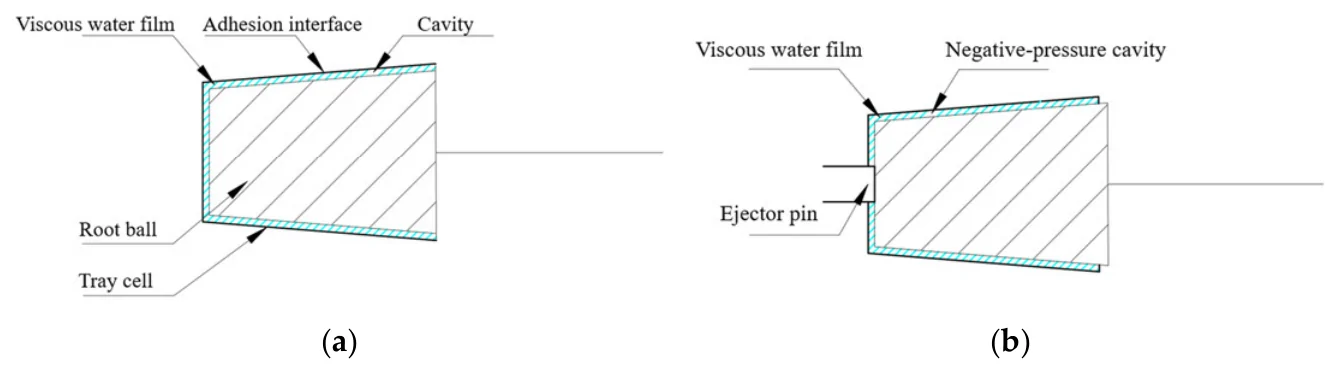
Schematic of adhesion during initial ejection: (**a**) before ejection; (**b**) after ejection.

Before ejection began, water films, pores, and localized regions of direct contact were present at the root ball–tray cell interface (Figure 4a). Hydrogen bonding and van der Waals forces acted across discontinuous water films, and the viscous force $F_{s}$ of these films contributed to the adhesive force $F_{t}$ [20,21]. A small ejector-pin displacement stretched the films, altered pore volume, and disturbed the adhesive equilibrium (Figure 4b). Detachment resistance was therefore represented by the combined effects of adhesion and local friction.

Ejector-pin loading displaced the root ball relative to the tray-cell sidewall, enlarging the interfacial cavities and generating suction from the resulting negative pressure. The adhesive force $F_{t}$ was expressed as the sum of the water-film viscous force $F_{s}$ and cavity-suction force $F_{n}$:

(5) $$F_{t}=F_{s}+F_{n}=\sum_{i}\frac{3\eta \pi R_{i}^{4}}{4\Delta t_{a}}\left(\frac{1}{h_{1}^{2}}-\frac{1}{h_{2}^{2}}\right)+\sum_{j}p_{kj}s_{pj}$$

where η is the soil-water viscosity (Pa s); $R_{i}$ is the water-film radius at interface i (m); $\Delta t_{a}$ is the separation time (s); $h_{1}$ is the initial separation between the substrate and the tray-cell sidewall (m); $h_{2}$ is the displacement of the substrate relative to the tray-cell sidewall (m); $p_{kj}$ is the negative pressure in cavity j (MPa); $s_{pj}$ is the cross-sectional area of cavity j (m^2^).

Root development and moisture content also affected the extent of direct contact with the tray-cell sidewall. Sliding friction $F_{f}$ therefore acted in addition to adhesion $F_{t}$, and the total short-duration resistance to detachment $F_{N}$ was:

(6) $$F_{N}=F_{t}+F_{f}=F_{s}+F_{n}+\sum_{k}\mu F_{nk}$$

where μ is the coefficient of friction between the root ball and the tray-cell sidewall; $F_{nk}$ is the normal force exerted by the root ball on the tray-cell sidewall at location k.

Because moisture content simultaneously affects water-film adhesion, cavity pressure, and contact friction, its effect on detachment may be nonlinear. Moisture content was therefore treated as an experimental factor rather than as a basis for estimating unmeasured interfacial parameters.

**Figure 5:**
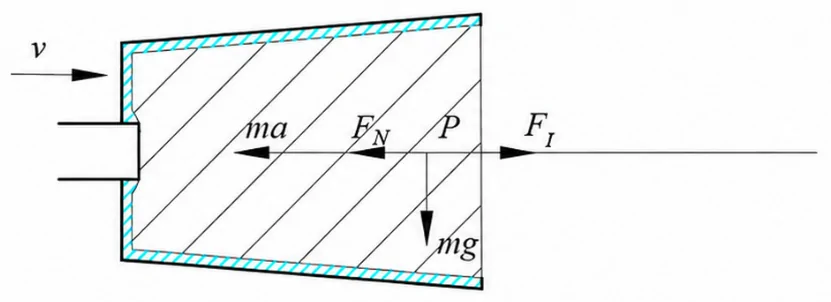
Force analysis of the root ball during the initial ejection stage.

During initial ejection, an ejector pin moving at v displaced a seedling of mass m over the short interval $t_{I}$ (Figure 5). Assuming constant pin velocity while the seedling velocity increased from 0 to v, Newton’s second law gives the following relation for the impact:

(7) $$F_{I}=ma+F_{N}=m\frac{v}{\Delta t_{I}}+F_{N}$$

Mechanical damage during high-speed transplanting depends on impact load, stem deformation, and root condition [22,23]. Because Equation (7) relates picking frequency to pin velocity and impact, picking frequency was included as an experimental factor.

(2) Second stage: Ejection-induced sliding

**Figure 6:**
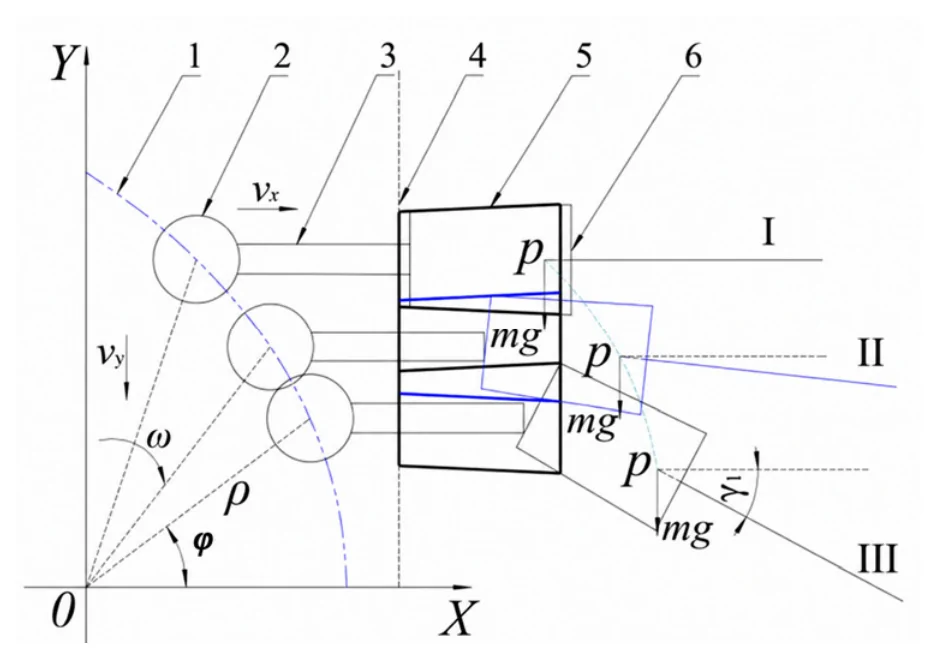
Schematic of the ejection-induced sliding stage. 1. Seedling-picking cam profile; 2. ejector-pin mounting shaft; 3. ejector pin; 4. tray-cell trajectory; 5. tray cell; 6. rice pot seedling.

The ejection-induced sliding stage began when the adhesive interface between the root ball and tray cell failed and ended when the ejector pin separated from the root ball. Positions I–III (Figure 6) describe the accompanying changes in center-of-mass position and edge contact.

The motion of the ejector pin was characterized by resolving its velocity into X– and Y–components using the polar equation of the cam return profile:

(8) $$\left\{\begin{array}{l} v_{x}=\frac{d}{dt}\left[\frac{114\Delta \varphi cos\left(\Delta \varphi \right)}{\pi sin\left(\Delta \varphi \right)}\right]\\ v_{y}=\frac{d}{dt}\left[\frac{114\Delta \varphi sin\left(\Delta \varphi \right)}{\pi sin\left(\Delta \varphi \right)}\right]\\ \Delta \varphi =\omega t \end{array}\right.$$

where $v_{x}$ is the horizontal component of ejector-pin velocity (m/s); $v_{y}$ is the vertical component of ejector-pin velocity (m/s); Δφ is the angle swept during time t by the line joining the pin to center O (°); ω is the mechanism angular velocity (rad/s).

Equation (8) yields:

(9) $$\left\{\begin{array}{l} v_{x}=\frac{114\omega \left[tan\left(\omega t\right)-\omega t{sec}^{2}\left(\omega t\right)\right]}{\pi {tan}^{2}\left(\omega t\right)}\\ v_{y}=\frac{114\omega }{\pi } \end{array}\right.$$

Numerical evaluation over positions I–III showed that the seedling initially underwent near-translational motion, after which its center of mass passed beyond the cell boundary, and the root ball tilted about the lower edge. At position III, the root-ball end reached the critical position at the upper cell edge, marking the onset of in-cell rotation.

(3) Third stage: In-cell rotation

**Figure 7:**
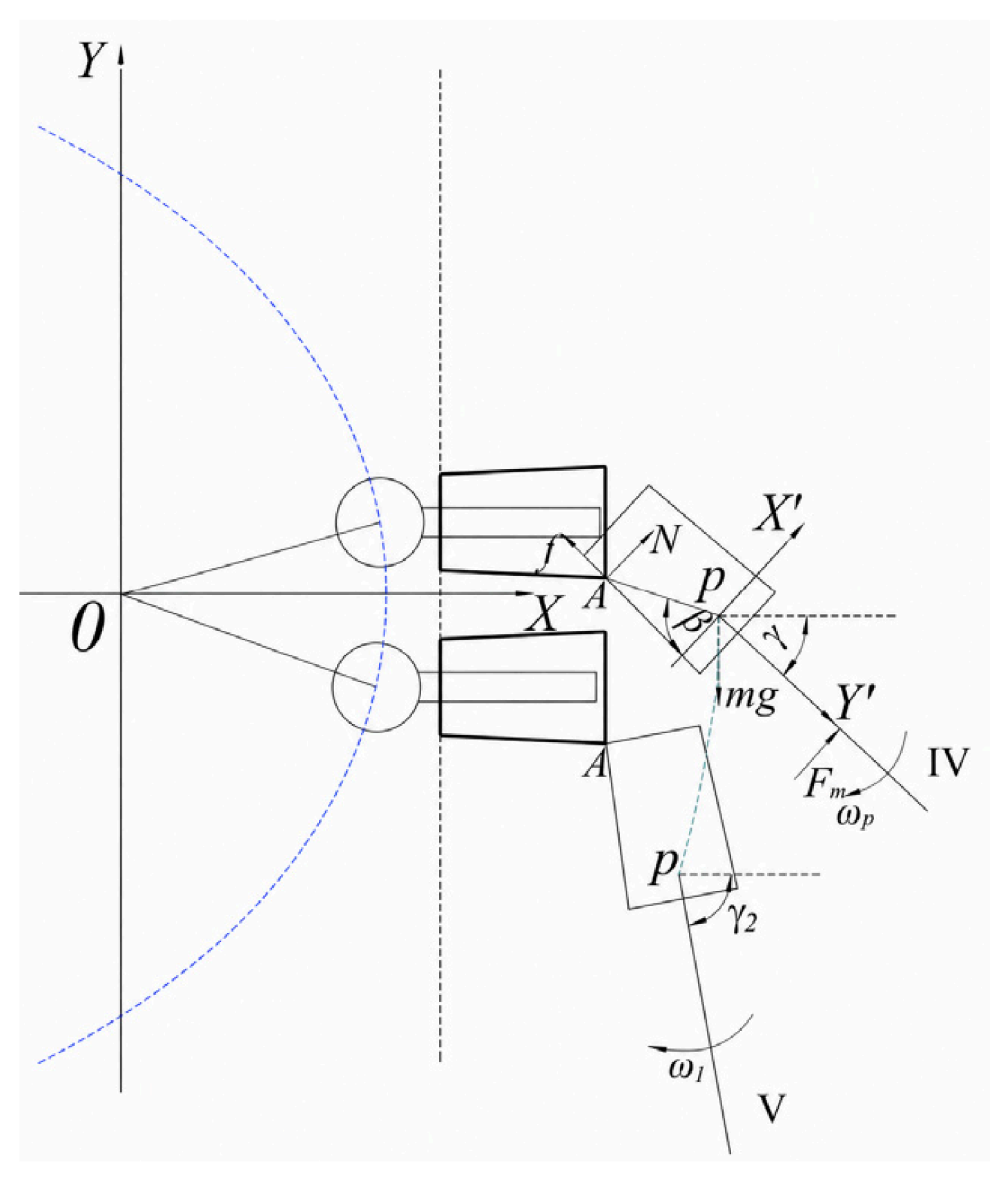
Schematic of the in-cell rotation stage.

In-cell rotation began when the root ball cleared the upper cell edge and continued until tray release, while the lower root-ball sidewall remained in contact with the lower cell edge (Figure 7).

The seedling rotated about A while its shoot slid along the seedling-separating plate. At position III, for picking frequencies of 4–8 s^−1^, Equation (9) gave a horizontal ejector-pin velocity $v_{x}$ of 0.03–0.05 m·s^−1^. Because this velocity was small relative to the gravity-driven motion of the seedling, direct action of the pin was neglected thereafter. A relative $X^{\prime }pY^{\prime }$ coordinate system was established with its origin at p. At position IV, the equation of motion was:

(10) $$\left\{\begin{array}{l} m\frac{d^{2}X^{\prime }}{dt^{2}}=N+F_{m}-mgcos\;\gamma \\ m\frac{d^{2}Y^{\prime }}{dt^{2}}=mgsin\;\gamma -f\\ I_{p}\frac{d^{2}\gamma }{dt^{2}}=fl_{f}+Nl_{N}-F_{m}l_{F_{m}}\\ f=\mu N \end{array}\right.$$

where γ is the seedling angle (°); f is the frictional resistance between the pot body and the tray cell (N); N is the reaction force acting between the pot body and the tray cell (N); $F_{m}$ is the frictional resistance between the shoot and the seedling-separating plate (N); $l_{f}$ is the moment arm of friction force f (m); $l_{N}$ is the moment arm of support reaction N (m); $l_{F_{m}}$ is the moment arm of friction force $F_{m}$ (m); $I_{p}$ is the moment of inertia about p (kg m^2^); μ is the coefficient of friction between the rice pot seedling and the tray cell.

Taking p as the reference point and projecting the relative-velocity relation onto the $X^{\prime }$–axis gives:

(11) $$\left\{\begin{array}{l} a_{MX^{\prime }}=\frac{d^{2}X^{\prime }}{dt^{2}}-l_{Ap}\alpha cos\;\gamma +l_{Ap}\omega_{p}^{2}cos\;\beta \\ l_{Ap}=\sqrt{l_{N}^{2}+l_{f}^{2}} \end{array}\right.$$

where $l_{Ap}$ is the distance from point A to the center of mass p (m); α is the instantaneous angular acceleration of the rice pot seedling (rad/s^2^); $\omega_{p}$ is the instantaneous angular velocity of the rice pot seedling (rad/s); β is the angle between Ap and the $X^{\prime }$–axis (°).

Because displacement in the $X^{\prime }$–direction was zero during tray feeding, $\vec{a_{MX^{\prime }}}=0$. Combining Equations (10) and (11) gives:

(12) $$\frac{d^{2}X^{\prime }}{dt^{2}}-l_{Ap}\alpha cos\;\gamma +l_{Ap}\omega_{p}^{2}cos\;\beta =0$$

(13) $$N=\frac{mI_{p}\left(gcos\;\gamma -l_{Ap}\omega_{p}^{2}cos\;\beta \right)-I_{p}F_{m}-mF_{m}l_{F_{m}}l_{Ap}cos\;\gamma }{I_{p}-ml_{Ap}cos\;\gamma \left(\mu l_{f}+l_{N}\right)}$$

At the critical tray-release state (position V), N=0; Equation (13) therefore reduces to Equation (14).

(14) $$\omega_{1}=\sqrt{\frac{mI_{p}gcos\;\gamma_{2}-I_{p}F_{m}-mF_{m}l_{F_{m}}l_{Ap}cos\;\gamma_{2}}{mI_{p}l_{Ap}cos\;\beta_{2}}}$$

where $\omega_{1}$ is the seedling angular velocity at the critical tray-release position (rad/s); $\gamma_{2}$ is the angle between the seedling and the horizontal at that instant (°).

$F_{m}$ denotes the frictional resistance between the shoot and the seedling-separating plate. As seedling age increases, shoot length, fresh mass, morphology, moment of inertia, and contact conditions change, affecting the rotational state at complete release. Because transient contact forces and angular motion at release were not measured, the release angular velocity could not be determined directly. Seedling age was therefore included as an experimental factor.

(4) Fourth stage: Post-release rotation

**Figure 8:**
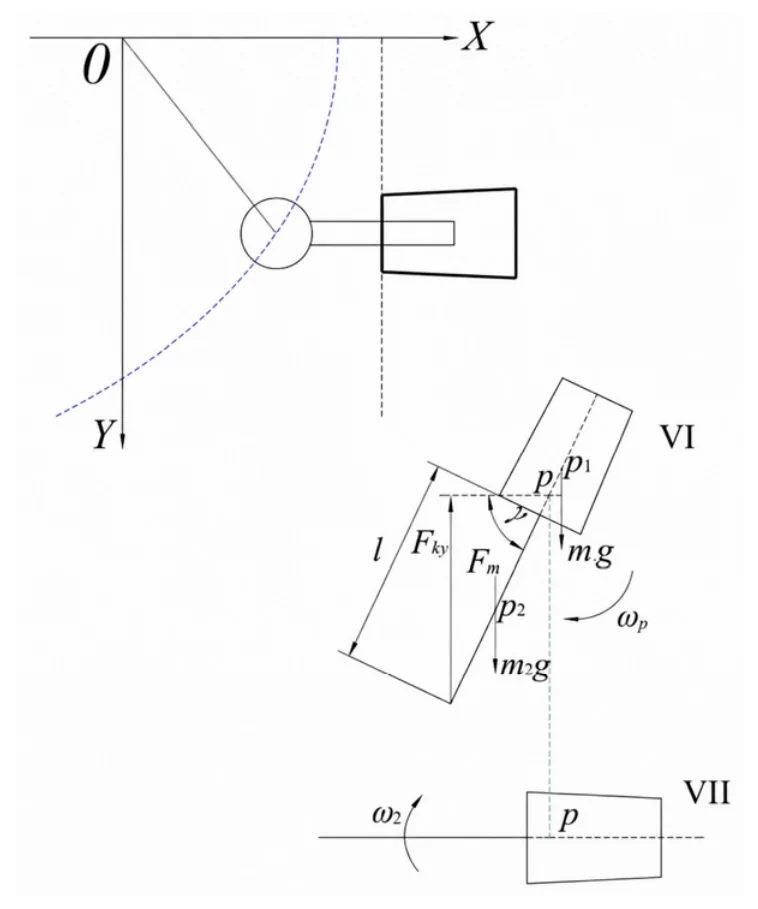
Schematic of the post-release rotation stage.

After the root ball lost contact with the tray cell at position V, the seedling continued to rotate about its center of mass as it fell toward the root-ball-leading, shoot-trailing orientation. Contact between the shoot and the seedling-separating plate could persist briefly until complete separation (Figure 8).

At position VI, the equations governing seedling motion were:

(15) $$\left\{\begin{array}{l} M_{ky}=F_{ky}l_{p2}cos\;\gamma \\ M_{p1}=m_{1}gl_{p1}cos\;\gamma \\ M_{p2}=m_{2}gl_{p2}cos\;\gamma \\ M_{m}=F_{m}l_{p2}cos\;\gamma \\ I_{p}\frac{d^{2}\gamma }{dt^{2}}=M_{p2}-M_{p1}-M_{m}-M_{ky} \end{array}\right.$$

where $F_{ky}$ is the total vertical aerodynamic drag, represented by an equivalent resultant acting at the shoot center of mass $p_{2}$ (N); $M_{ky}$ is the moment generated by $F_{ky}$ (N m); $m_{1}$ is the mass of the root ball (kg); $m_{2}$ is the mass of the shoot (kg); $M_{p1}$ is the gravitational moment of the root ball about p (N m); $M_{p2}$ is the gravitational moment of the shoot about p (N m); $l_{p1}$ is the distance from the root-ball center of mass $p_{1}$ to the overall seedling center of mass p, $l_{p1}=\;L_{p}-\;L_{1}$ (m); $l_{p2}$ is the distance from the shoot center of mass $p_{2}$ to the overall seedling center of mass p, $l_{p2}=\;L_{2}-\;L_{p}$ (m); $M_{m}$ is the moment generated by the resistance force $F_{m}$ (N m).

Equation (15) yields

(16) $$\frac{d^{2}\gamma }{dt^{2}}=\frac{d\omega_{p}}{d\gamma }\frac{d\gamma }{dt}=\omega_{p}\frac{d\omega_{p}}{d\gamma }$$

(17) $$\frac{1}{2}\omega_{p}^{2}=-\frac{1}{I_{p}}\left[\left(m_{1}gl_{p1}+F_{m}l_{p2}+F_{ky}l_{p2}-m_{2}gl_{p2}\right)sin\;\gamma \right]+C_{0}$$

where $C_{0}$ is the constant of integration.

The upright root-ball-down, shoot-up orientation $\left(\gamma =\frac{\pi }{2},\omega_{p}=0\right)$ was taken as the normalized theoretical reference state. Substitution into Equation (17) gives:

(18) $$C_{0}=\frac{m_{1}gl_{p1}+F_{m}l_{p2}+F_{ky}l_{p2}-m_{2}gl_{p2}}{I_{p}}$$

At the root-ball-leading, shoot-trailing orientation (position VII), γ=0. Combining Equations (17) and (18) gives:

(19) $$\omega_{2}=\sqrt{2C_{0}}=\sqrt{\frac{2(m_{1}gl_{p1}+F_{m}l_{p2}+F_{ky}l_{p2}-m_{2}gl_{p2})}{I_{p}}}$$

Because the interval ${\Delta t}_{r}$ between release at position V and attainment of the target orientation at position VII was short, angular acceleration was assumed constant. Thus:

(20) $$\Delta t_{r}=\frac{2\left(2\pi -\gamma_{2}\right)}{\omega_{1}+\omega_{2}}$$

Assuming uniformly accelerated vertical motion under the equivalent mean aerodynamic drag, the reorientation height was

(21) $$h=\frac{1}{2}(g-\frac{F_{ky}}{m_{1}+m_{2}})\Delta t_{r}^{2}=\frac{2(g-\frac{F_{ky}}{m_{1}+m_{2}}){\left(2\pi -\gamma_{2}\right)}^{2}}{{\left(\omega_{1}+\omega_{2}\right)}^{2}}$$

Instantaneous aerodynamic and contact forces, together with the angular motion at release, were not measured. Equations (15)–(21) were therefore not fitted numerically to the image data; they provide theoretical estimates and reference values only. High-speed photography was used instead to assess the four-stage orientation sequence qualitatively.

### 2.3. High-Speed Photography Bench Test of Post-Ejection Reorientation in Rice Pot Seedlings

Zhongjiazao 17 seedlings were grown in Yameike D448P trays using a substrate from Shenjiang Agricultural Development Co., Ltd. (Nanchang, China). Each tray was sown with a single centered row in accordance with NY/T 1607-2008.

**Figure 9:**
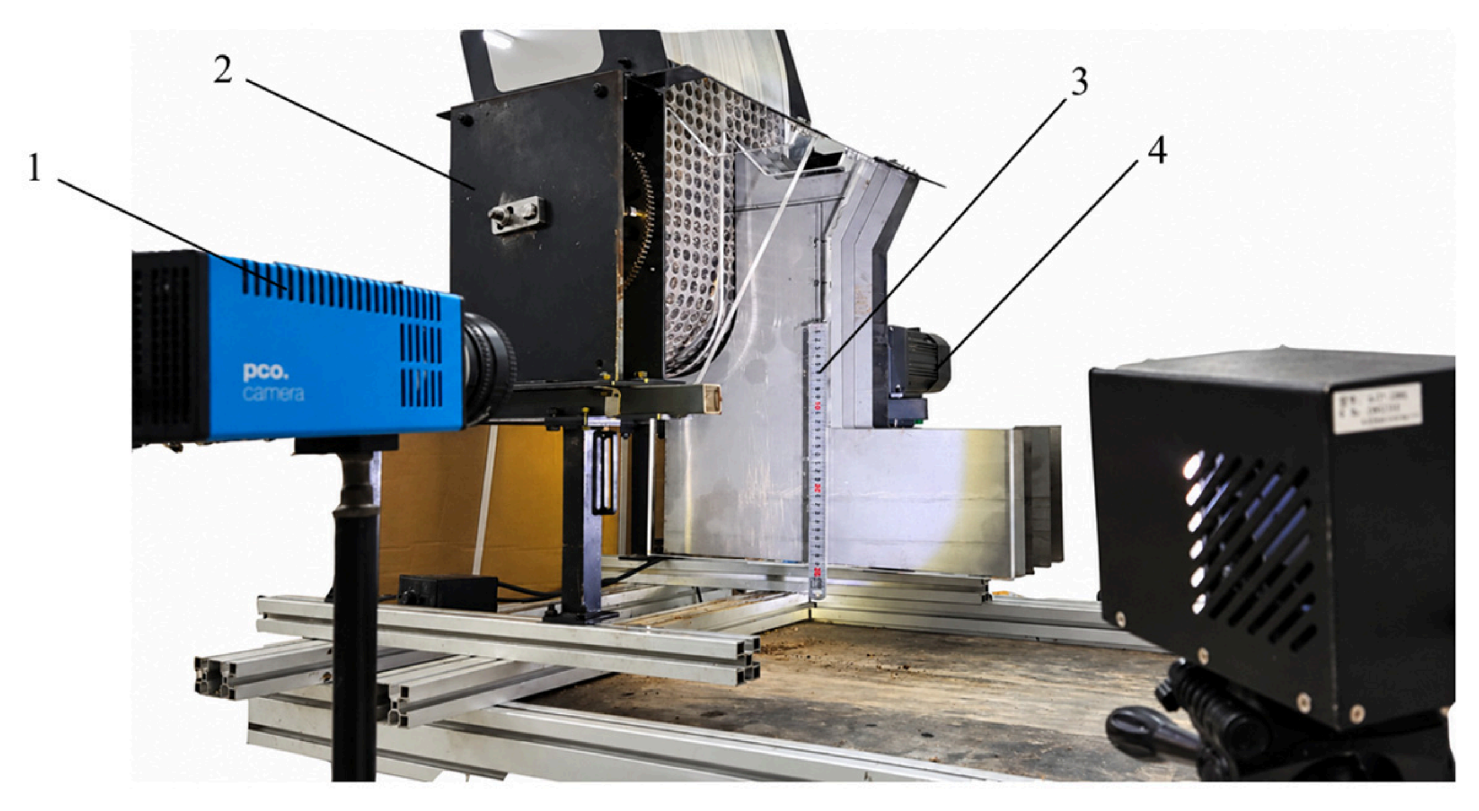
High-speed photography test bench: 1. High-speed camera; 2. seedling-picking device; 3. reference ruler; 4. drive motor.

The test bench consisted of the picking device, drive motor, speed controller, pco.1200/hs camera (PCO AG, Kelheim, Germany), and vertical reference ruler (Figure 9). A fixed side-view camera recorded at 500 frames s^−1^ with a resolution of 1280 × 1024 pixels, corresponding to an interframe interval of 2 ms. At the highest seedling-picking frequency of 8 s^−1^, the shortest observed durations from ejection-induced sliding to complete release and from complete release to the first attainment of the target orientation were 125 ms (approximately 63 frames) and 256 ms (128 frames), respectively, indicating sufficient temporal resolution for stages 2–4. The camera image plane was aligned parallel to the principal motion plane, and its optical axis was normal to that plane. The two parallel seedling-separating plates were aligned parallel to the camera image plane and covered the entire region used for orientation assessment. The spacing between the plates was 18 mm, whereas the upper diameter of the root ball was approximately 15 mm. The plates therefore guided the falling seedlings and constrained large out-of-plane displacement and rotation but did not necessarily eliminate them. Because only a single side view was recorded, residual out-of-plane rotation and its contribution to angular uncertainty could not be quantified from the available images. Accordingly, angular measurements derived from these images were interpreted as projected angles in the principal motion plane. A one-dimensional spatial scale was established from a known ruler length and the corresponding pixel count. Reorientation height was then calculated from the vertical pixel difference between complete release and the first attainment of the target orientation. No obvious perspective distortion was observed in the recorded region. Additional instruments included a 0.02 mm vernier caliper, DLY-2301D tachometer (Delixi Electric Ltd., Yueqing, China; 0.1 r min^−1^), DH-190 moisture analyzer (Shanghai Danhao Electronic Technology Co., Ltd., Shanghai, China), an illuminator, and MTB 3000D electronic balance (Shenzhen Mei Foo Electronics Co., Ltd., Shenzhen, China; readability: 0.01 g).

### 2.4. Experimental Factors and Box–Behnken Design

Root-ball moisture content, seedling age, and picking frequency were selected to represent interfacial condition, mass and contact differences, and pin-motion input, respectively. The root-ball moisture-content levels of 40%, 50%, and 60% were selected based on Reference [19]. Before each run, the seedlings were thoroughly watered and allowed to dry naturally. Root-ball moisture content was measured in five randomly selected seedlings using a DH-190 halogen moisture analyzer, and their mean represented the tray moisture content. Natural drying or rewatering followed by drying was repeated until the mean reached the target level within ±2 percentage points, after which the high-speed photography experiment was conducted immediately. Seedling age was set to 2.5, 3.0, or 3.5 leaves, and picking frequency to 4, 6, or 8 s^−1^. A three-factor Box–Behnken design with 17 runs, including five center-point runs, was used (Table 2).

### 2.5. Response Variable Definitions and Calculations

**Figure 10:**
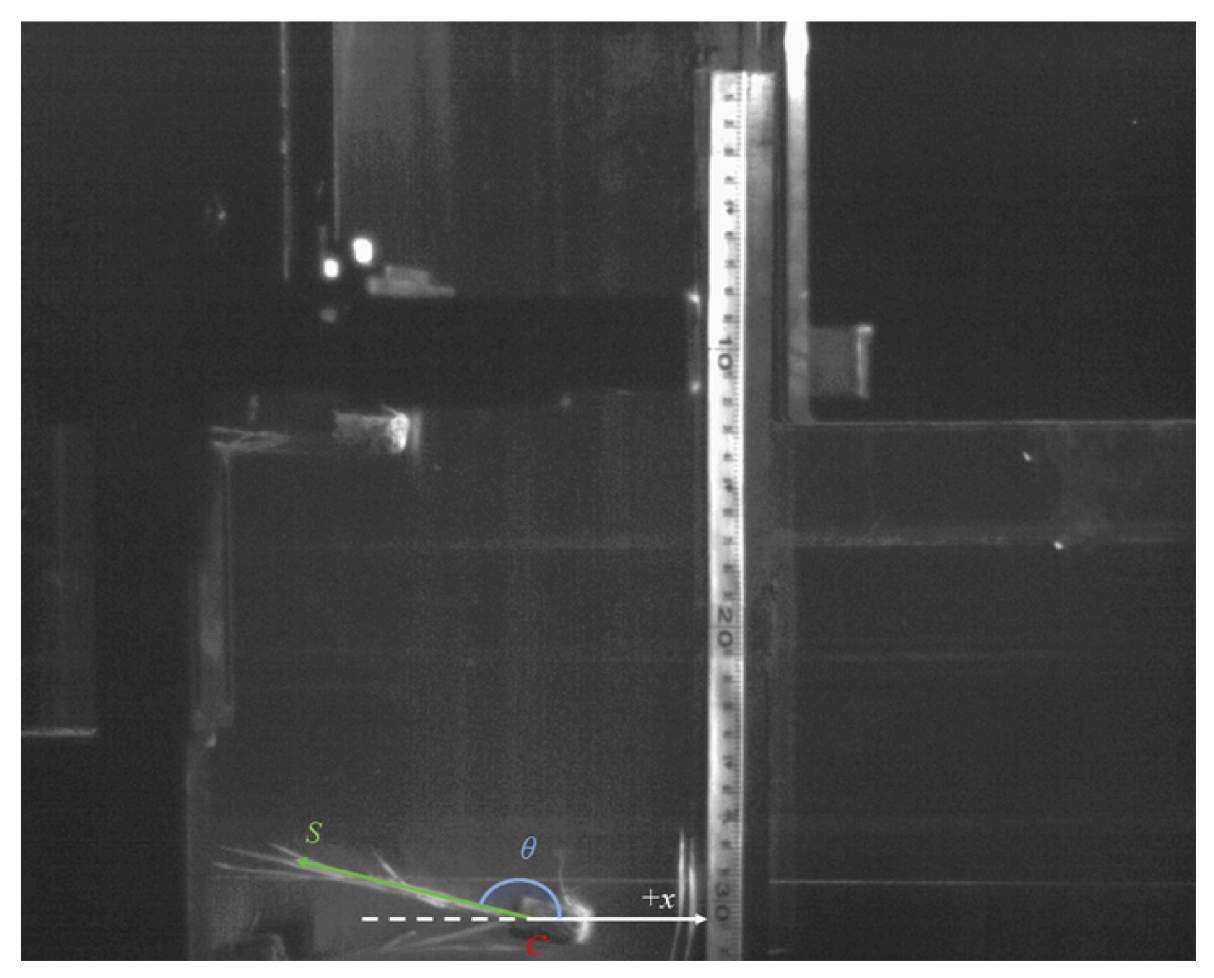
Definition of the projected rotation angle. C, root-ball centroid; S, shoot reference point; +x, positive horizontal direction; θ, projected rotation angle.

Reorientation success rate $F_{c}$: A reorientation was considered successful when the seedling attained the root-ball-leading, shoot-trailing orientation within the prescribed drop distance and its projected rotation angle θ was between 165° and 195°. The angle was manually measured frame by frame from the side-view high-speed images using ImageJ (version 1.54t; National Institutes of Health, Bethesda, MD, USA) and was defined as the angle from the positive horizontal direction to the line connecting the root-ball centroid C and the shoot reference point S, as shown in Figure 10. The exact target orientation corresponded to θ = 180°. The camera optical axis was normal to the principal motion plane, and the separating plates constrained out-of-plane motion. All images were evaluated by the same observer using identical reference-point definitions and measurement criteria. Formal intra-observer repeatability and inter-observer agreement were not assessed.

Each experimental run comprised 32 ejection attempts. A successfully ejected seedling was defined as a seedling whose root ball completely separated from the tray cell and entered the observable drop region without remaining lodged in the tray. Consequently, the number of successfully ejected seedlings, $N_{q}$, ranged from 28 to 32 among the 17 runs. Reorientation success rate was calculated using $N_{q}$, rather than the 32 attempted ejections, as the denominator. No failed ejection was replaced within a run. $F_{c}$ was calculated as the ratio of successfully reoriented seedlings to successfully ejected seedlings:

(22) $$F_{c}=\frac{N_{c}}{N_{q}}\times 100\%$$

where $N_{c}$-The number of seedlings that reoriented successfully; $N_{q}$-The total number of seedlings ejected successfully.

Reorientation height $H_{f}$: For each successfully reoriented seedling, reorientation height was defined as the vertical distance from complete release to the first occurrence of the target orientation. $H_{f}$ was calculated as the arithmetic mean of the reorientation heights of the $N_{c}$ successfully reoriented seedlings.

## 3. Results

### 3.1. Results and Analysis of the High-Speed Photography Bench Test of Post-Ejection Reorientation

**Figure 11:**
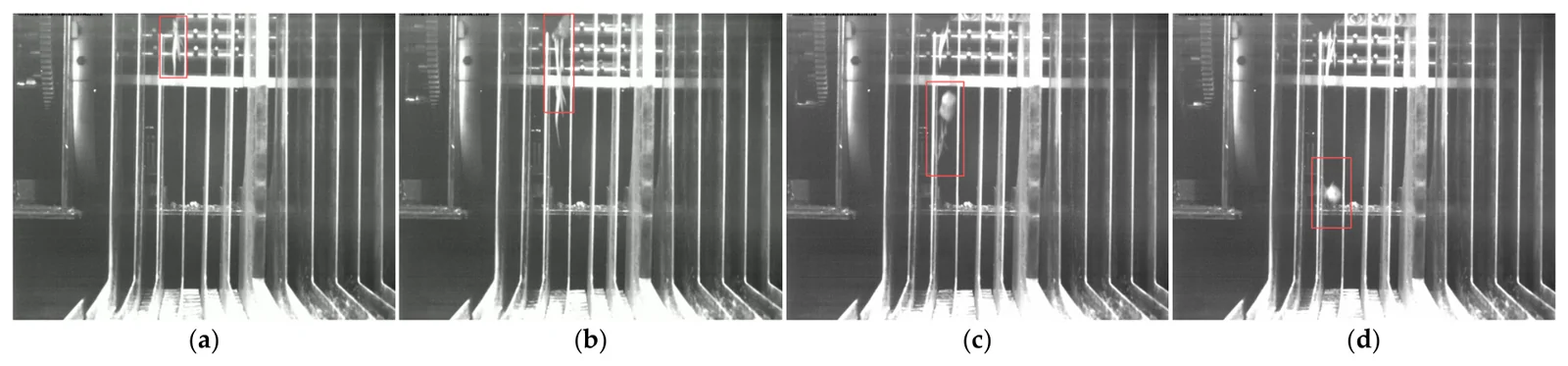
High-speed photographic sequence from the bench test: (**a**) Ejection-induced sliding stage; (**b**) in-cell rotation stage; (**c**) post-release rotation stage; (**d**) target orientation achieved.

As shown in Figure 11, high-speed photography captured the observable motion from ejection-induced sliding (stage 2), through in-cell rotation (stage 3) and post-release rotation (stage 4), to completion of the target orientation. The microscale initial-ejection stage (stage 1) could not be reliably resolved because it occurred over a short duration and was partly obscured by the tray cell. During stages 2–4, the seedling underwent sliding and tilting within the tray cell, rotation during release, and subsequent formation of the root-ball-leading, shoot-trailing orientation. This observed sequence was qualitatively consistent with the theoretical motion analysis but did not constitute quantitative validation of the initial ejection process, release angle, release angular velocity, or transient contact forces.

The experimental results are presented in Table 3. Quadratic response-surface models were fitted to the 17 runs in Design-Expert 11, using moisture content, seedling age, and picking frequency as factors and run-level reorientation success rate and mean reorientation height as responses. ANOVA was used to evaluate the overall models, main effects, and interaction effects; the fitted equations are:

(23) $$Y_{1}=86.36+1.94X_{1}-3.23X_{2}-1.59X_{3}-1.64X_{1}X_{2}-2.23X_{1}X_{3}+0.29X_{2}X_{3}-0.65{X_{1}}^{2}\;+1.45{X_{2}}^{2}-0.92{X_{3}}^{2}$$

(24) $$Y_{2}=39.44+2.51X_{1}+1.31X_{2}+2.02X_{3}-1.03X_{1}X_{2}+0.5X_{1}X_{3}-1.1X_{2}X_{3}-1.36{X_{1}}^{2}\;+0.29{X_{2}}^{2}-1.03{X_{3}}^{2}$$

Subsequently, variance analysis was performed on the regression equations, with results detailed in Table 4 and Table 5.

**Figure A1:**
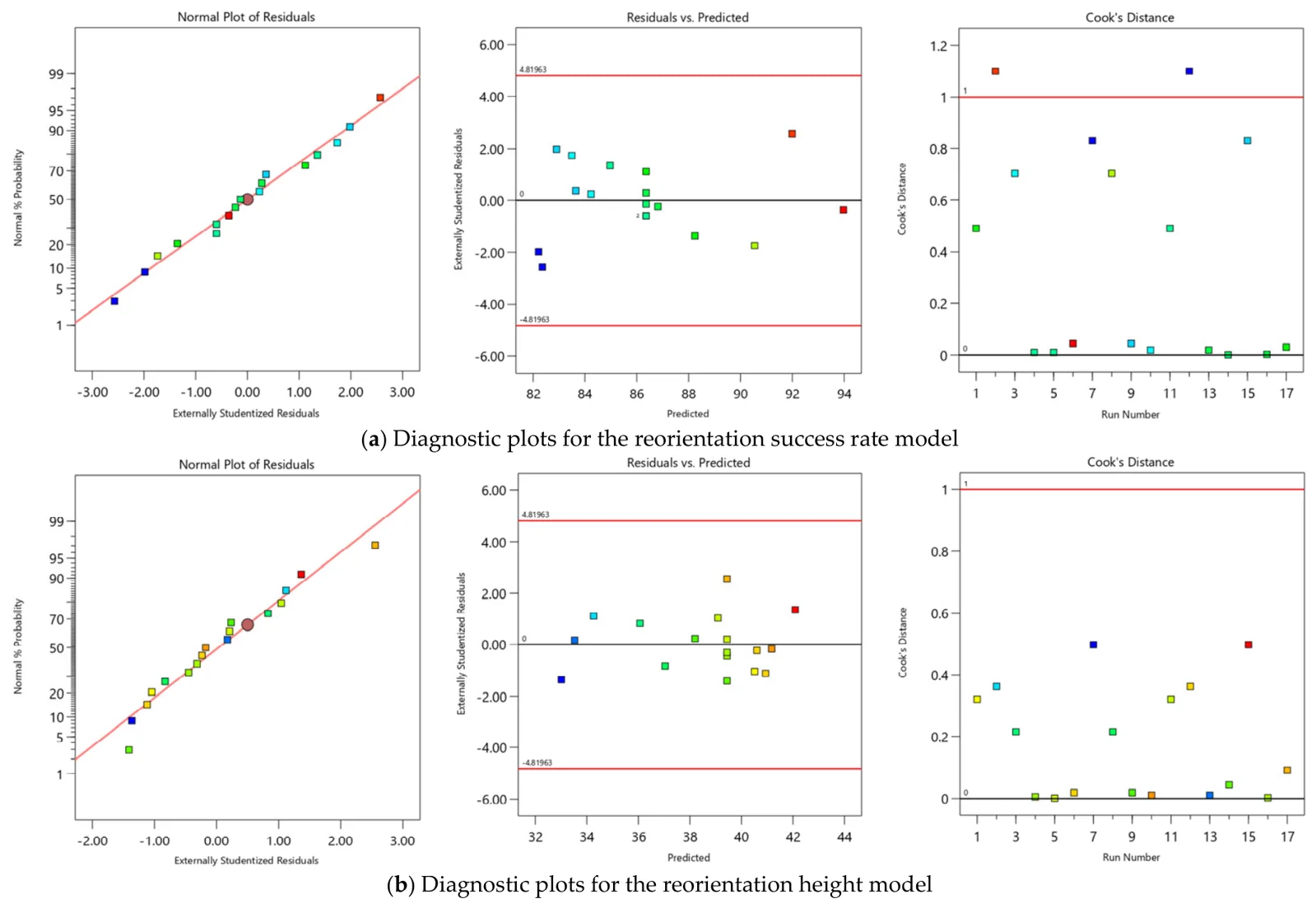
Diagnostic plots for the quadratic response-surface models.

Both quadratic models were significant, whereas the lack-of-fit terms were nonsignificant (Table 4 and Table 5). Model diagnostics are summarized in Table 6, with detailed residual diagnostics and influential-point assessments provided in Appendix A (Figure A1). Because goodness of fit does not establish predictive performance outside the experimental domain, all interpretation and optimization were restricted to the tested factor ranges.

All three factors significantly affected $Y_{1}$ (Table 4), and the F-value ranking was $X_{2}>X_{1}>X_{3}$. The interactions $X_{1}X_{2}$ and $X_{1}X_{3}$ were significant. The $R^{2}=0.9499$ and adjusted $R^{2}=0.8855$ values indicated good model fit, whereas the predicted $R^{2}=0.3727$ suggested limited predictive capability beyond the fitted data. The model was therefore used only within the experimental range. Adequate precision was 13.2371 (>4).

All three factors significantly affected $Y_{2}$ (Table 5), and the F-value ranking was $X_{1}>X_{3}>X_{2}$. The interactions $X_{1}X_{2}$ and $X_{2}X_{3}$ were significant. The $R^{2}$, adjusted $R^{2}$, and predicted $R^{2}$ values were 0.9644, 0.9185, and 0.7696, respectively. Adequate precision was 14.8578 (>4), supporting response analysis and optimization within the experimental range.

**Figure 12:**
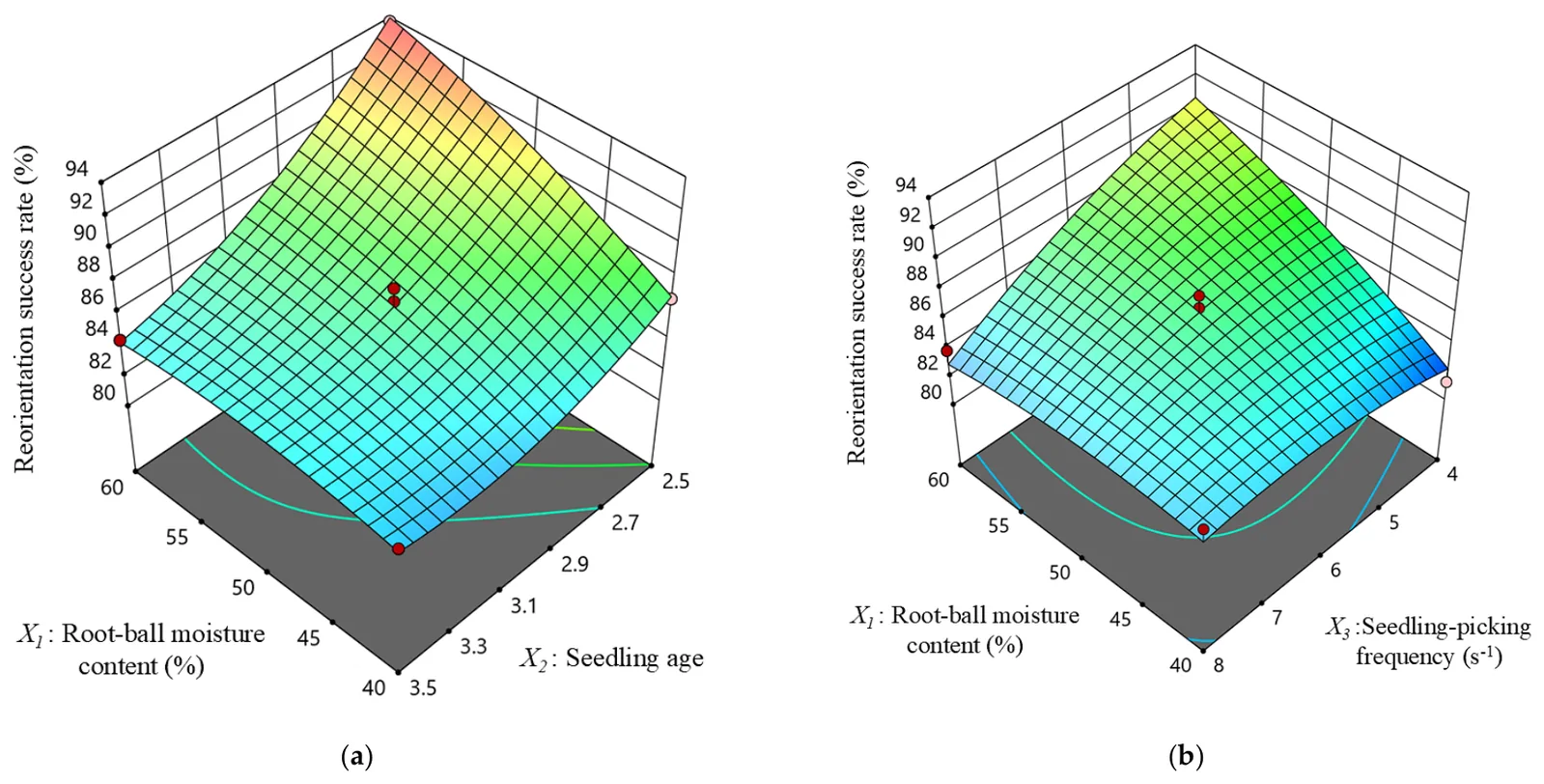
Response surfaces for the effects of interacting factors on reorientation success rate: (**a**) root-ball moisture content × seedling age; (**b**) root-ball moisture content × seedling-picking frequency.

**Figure 13:**
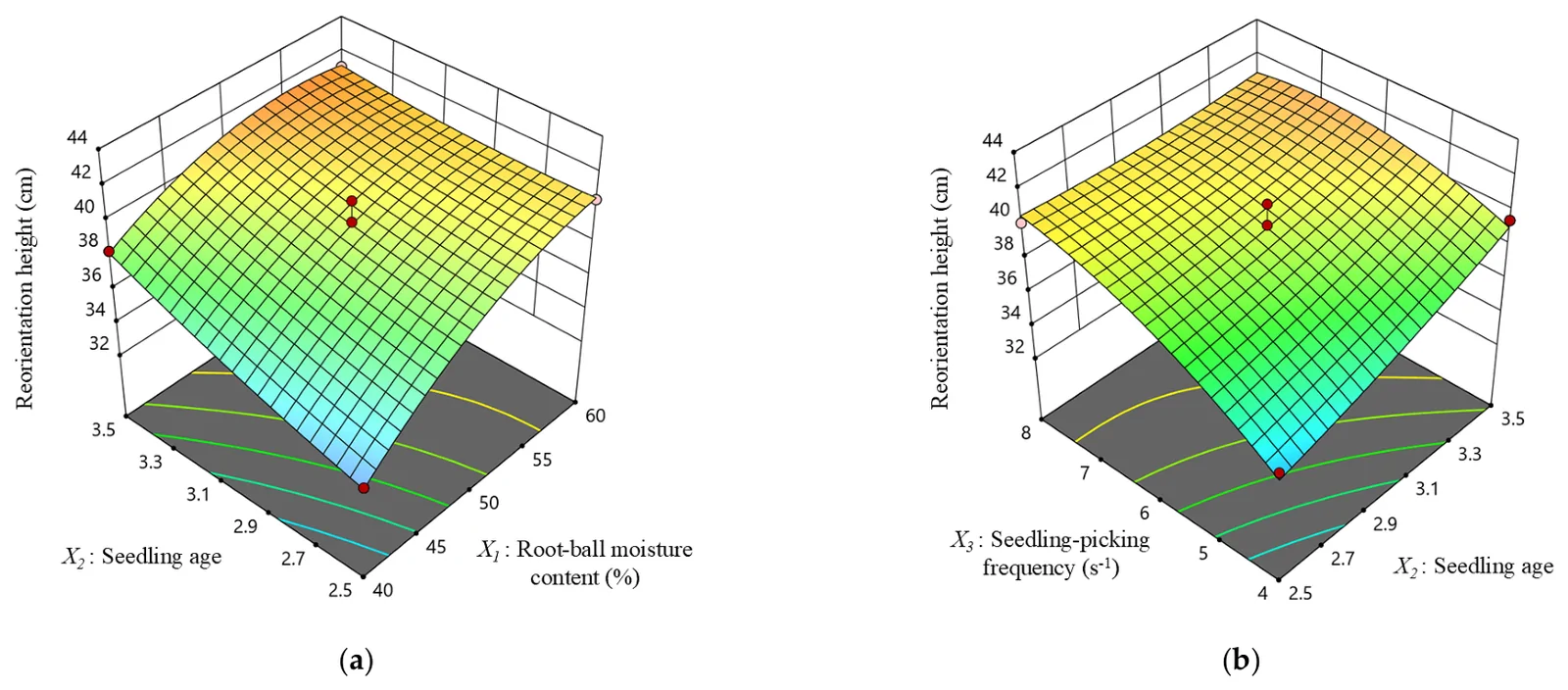
Response surfaces for the effects of interacting factors on reorientation height: (**a**) root-ball moisture content × seedling age; (**b**) seedling age × seedling-picking frequency.

Response surfaces for reorientation success rate $Y_{1}$ and reorientation height $Y_{2}$ were generated from the fitted regression models for the significant interactions (Figure 12 and Figure 13).

In Figure 12a, reorientation success rate generally increased with moisture content and decreased with seedling age, and the moisture–age interaction was significant. Moisture content modifies mass distribution, interfacial adhesion, and friction, whereas seedling age changes height, fresh mass, morphology, and contact with the seedling-separating plate. These age-related changes may alter the angular state at release, as suggested by Equation (14). Because release angular velocity was not measured, the equation is used as a mechanistic reference rather than as a quantitatively validated relationship. The influence of seedling mechanical properties on transplanting load and damage has also been reported [23]. Figure 12b shows that reorientation success rate generally decreased as picking frequency increased. Higher pin velocity and impact likely increased variability in the release state and reduced the stability of target-orientation formation.

In Figure 13a, reorientation height generally increased with moisture content and seedling age, and their interaction was significant. Changes in mass distribution, moment of inertia, interfacial condition, shoot dimensions, and plate contact may alter the angular state at release and the distance required to reach the target orientation. Figure 13b shows a significant age–frequency interaction and an overall increase in reorientation height as picking frequency increased. Under the idealized assumptions, Equations (7), (20) and (21) provide a theoretical reference indicating that higher pin velocity can increase inertial disturbance at release and the drop distance required for stable rotation.

To coordinate stable target-orientation formation with a compact layout for downstream automated handling, multiresponse optimization in Design-Expert 11 was used to maximize $Y_{1}$ and minimize $Y_{2}$ within the tested factor ranges.

(25) $$\left\{\begin{array}{c} \max Y_{1}(X_{1}{,X}_{2},X_{3})\\ \min Y_{2}(X_{1}{,X}_{2},X_{3})\\ 40\%\leq X_{1}\leq 60\%\\ 2.5\leq X_{2}\leq 3.5\\ 4\leq X_{3}\leq 8 \end{array}\right.$$

The numerical optimum was a root-ball moisture content of 53.14%, a seedling age of 2.5 leaves, and a seedling-picking frequency of 4 s^−1^. The corresponding quadratic-model estimates were a reorientation success rate $Y_{1}$ of 93.75% and a reorientation height $Y_{2}$ of 35.08 cm.

### 3.2. Optimal-Point Confirmation

Because root-ball moisture content could be controlled only within ±2 percentage points, the numerical optimum of 53.14% was converted to a practical target of 53%, maintained within 51–55%. The 95% prediction intervals associated with the numerical optimum were 90.09–97.42% for reorientation success rate and 32.56–37.60 cm for reorientation height. Bench confirmation was therefore conducted at the practical setting of 53% root-ball moisture content, maintained within ±2 percentage points, at the 2.5-leaf stage, and at 4 s^−1^. The measured reorientation success rate and reorientation height were 93.33% and 33.70 cm, respectively, corresponding to relative deviations of 0.45% and 3.94% from the model estimates. Both measured values fell within their respective 95% prediction intervals, supporting agreement between the observations and model estimates at the optimized setting within the experimental domain. The remaining differences may reflect variability among individual seedlings and local substrate moisture, as well as simplifications in the representations of the root ball–tray interface and shoot.

However, confirmation was conducted only at the model-predicted optimum and should therefore be interpreted as optimal-point confirmation rather than full model validation. It does not establish predictive accuracy at other locations within the design space. Although the reorientation-success model showed a relatively high adjusted $R^{2}$ of 0.8855, its predicted $R^{2}$ was only 0.3727, indicating limited leave-one-run-out prediction stability. Therefore, the response surfaces and numerical optimum were interpreted as within-domain estimates rather than as evidence of general predictive capability. Predictive performance at other independent non-design points within the experimental domain remains unverified. Future validation should include non-center confirmation points and independent seedling batches. Evaluation beyond the investigated factor ranges would constitute extrapolation and was outside the scope of this bench study. Accordingly, the practical recommendation applies only within the tested factor ranges and should not be interpreted as a global optimum.

## 4. Discussion

This study linked the ejection action of a cam-driven continuous picking system to post-ejection reorientation of rice pot seedlings. Initial ejection involves microscale changes in interfacial water films and cavities and very small displacement at the root ball–tray cell interface. Because these brief events were partly obscured by the tray cell, they could not be reliably resolved by high-speed photography. The recordings instead captured ejection-induced sliding, in-cell rotation, and post-release rotation. Their sequence, culminating in the root-ball-leading, shoot-trailing orientation, was qualitatively consistent with the theoretical analysis. This supports the four-stage framework as a process-based explanation of how continuous picking generates the initial state for downstream automated handling but does not quantitatively validate the initial ejection process, release angle, release angular velocity, or transient contact forces.

Previous studies have mainly examined seedling trajectories and orientations during free fall, passage through guide tubes, or throwing operations [15,16,17]. By examining how the motion state develops before and during tray release, the present study extends this analysis upstream to the interface between automated picking and post-release handling. A self-sequencing rice transplanter likewise identifies ordered delivery and adaptability to seedling morphology and substrate condition as equipment-design objectives [24]. The root-ball-leading, shoot-trailing orientation therefore provides a measurable target for coordinating picking with downstream conveying and planting. In precision agricultural mechanization, this framework translates biological and material variability, together with actuator input, into measurable process targets and supplies physical variables for future vision-based monitoring and feedback control. More consistent orientation may reduce misdelivery and collision in integrated systems, although this downstream effect remains to be verified.

Root-ball moisture content, seedling age, and seedling-picking frequency significantly affected reorientation success rate and reorientation height, but their importance differed between responses. The influence order was seedling age, moisture content, and frequency for reorientation success rate, but moisture content, frequency, and seedling age for reorientation height. Target-orientation formation and the vertical distance required are therefore related but distinct aspects of performance, reflecting interfacial detachment, mass distribution, shoot–plate contact, and ejector-pin motion.

Within the tested range, increasing root-ball moisture content generally increased both responses. Higher moisture increased fresh mass and shifted the center of mass slightly toward the root-ball base, potentially altering inertia and responses to contact and aerodynamic moments. It also affects water-film adhesion, cavity suction, and friction at the root ball–tray cell interface, thereby modifying detachment and release. For pepper plug seedlings, Zhang et al. similarly examined root-ball moisture content, seedling-picking frequency, and ejection height as coupled factors. Their selected combination of 45% moisture content, 60 plants min^−1^, and a 10 mm ejection height produced a 98% picking success rate and a 1.98% pot damage rate [25]. Although the crop and device differed, these findings support treating substrate condition and mechanical input as interacting determinants of seedling release. The significant moisture content × seedling age interactions for both responses and the moisture content × frequency interaction for reorientation success rate further show that moisture effects depend on seedling morphology and operating conditions.

As seedling age increased from the 2.5-leaf to the 3.5-leaf stage, height, fresh mass, and center-of-mass position changed. Longer shoots may destabilize shoot–plate contact, while changes in mass distribution and moment of inertia may reduce reorientation consistency. Rice seedling stems also exhibit nonlinear creep and stress-relaxation responses as applied stress and strain change [26]. These morphological and time-dependent changes may explain the lower reorientation success rate and greater reorientation height at later stages. This interpretation agrees with reports that seedling mechanical properties, root condition, and stem deformation affect loading and damage during high-speed transplanting [23]. However, because shoot–plate forces and release angular motion were not measured, this mechanism remains an interpretation based on force analyses and experimental trends.

Increasing seedling-picking frequency from 4 to 8 s^−1^ increased ejector-pin velocity and transient impact, potentially increasing dispersion in the release state. In a high-speed dual-row plug-seedling pick-up device, buffer optimization reduced peak end-effector acceleration by 39.4% [27]. Although its crop and mechanism differed, this result indicates that transient acceleration, rather than nominal cycle frequency alone, is important for stable, low-damage handling. This explains the observed decrease in reorientation success rate and increase in reorientation height. The significant seedling age × frequency interaction for reorientation height suggests that different seedling morphologies respond differently to the same mechanical inputs. Higher frequency may therefore improve theoretical operating efficiency but reduce orientation stability and require greater vertical drop space.

Numerical optimization yielded a root-ball moisture content of 53.14%, the 2.5-leaf stage, and 4 s^−1^. Because root-ball moisture content could be controlled only within ±2 percentage points, the numerical value of 53.14% was translated into a practical target of 53%, maintained within 51–55%. At the numerical optimum, the regression models estimated a reorientation success rate of 93.75% and a reorientation height of 35.08 cm, while bench confirmation at the practical setting yielded 93.33% and 33.70 cm, respectively. The small relative deviations support agreement between the observations and model estimates at the recommended combination within the experimental domain but do not establish predictive accuracy throughout the design space. The measured reorientation height provides a reference for allocating vertical space between the picking and downstream mechanisms, with a safety margin for individual variability. From a precision-agriculture perspective, this practical operating range converts substrate condition, plant developmental stage, and machine frequency into explicit control targets, reducing reliance on empirical adjustment. The recommendation applies only within the tested ranges and should not be interpreted as a global optimum.

Rice pot-seedling transplanting has been reported to promote rapid crop establishment and favorable yield performance [1,2]. The productivity relevance of the present work is therefore indirect but operationally important: repeatable release orientation and adequate drop space are upstream requirements for orderly transfer and consistent planting, which may help preserve seedling integrity and stand uniformity. However, neither field establishment nor grain yield was measured, and the optimized picking system should not be interpreted as having directly increased crop productivity. Technical limitations also remain. The initial ejection stage was not directly resolved, and the release angle, release angular velocity, root ball–tray cell forces, aerodynamic moment, and residual shoot–plate contact force were not measured. Equations (15)–(21) therefore provide theoretical estimates and reference values under idealized assumptions rather than quantitatively validated descriptions of the actual motion. The bench experiments used one rice cultivar, one tray type, and two-dimensional side-view imaging.

Future work should characterize initial ejection using dual-view or three-dimensional imaging and transient-contact measurements, and validate the models with independent seedling batches and non-center confirmation points. For transfer to field machinery, the recommended settings should be treated as monitored operating ranges rather than fixed constants. Root-ball moisture should be maintained near 53% (±2 percentage points), seedling variability addressed through screening or vision-based adjustment, and picking frequency regulated by closed-loop speed control. Machine motion, vibration, wind, tray occupancy, and downstream components may alter the release state. Therefore, the measured 33.70 cm reorientation height should guide vertical-space allocation with a safety margin. Integrated field tests should relate release orientation to conveying performance, collision damage, missing-plant rate, planting uprightness, establishment, and yield.

## 5. Conclusions

A root-ball-leading, shoot-trailing in-air reorientation method was developed for the picking-to-transfer interface of precision automated rice transplanting. Based on center-of-mass distribution and pin–seedling contact, the process was resolved into four stages: initial ejection, ejection-induced sliding, in-cell rotation, and post-release rotation. This four-stage description clarified how the release state required for downstream automated handling was formed and how the seedling subsequently attained the target orientation.

Root-ball moisture content, seedling age, and picking frequency significantly affected both reorientation success rate and reorientation height. The F-value rankings were age > moisture content > frequency for reorientation success rate and moisture content > frequency > age for reorientation height. Within the tested ranges, a practical root-ball moisture target of 53% (maintained within ±2 percentage points), the 2.5-leaf stage, and 4 s^−1^ were recommended. Bench confirmation yielded a reorientation success rate of 93.33% and a reorientation height of 33.70 cm. Both values fell within their respective 95% prediction intervals, supporting agreement with the model estimates at the recommended combination within the experimental domain. The four-stage framework and recommended operating window provide a basis for parameter matching, vertical-space design, and release-orientation management in precision automated transplanting. By improving controllability at the picking-to-transfer interface, these findings support the consistent application of rice pot-seedling transplanting, a crop-establishment approach with reported productivity benefits. Direct effects on stand establishment and grain yield were not evaluated and require validation using an integrated field system.

## Figures and Tables

**Table 1:** Physical properties of rice pot seedlings.

Root-Ball Moisture Content (%)	2.5-Leaf Stage		3.0-Leaf Stage		3.5-Leaf Stage	
	Center of Mass Position (mm)	Fresh Mass (g)	Center of Mass Position (mm)	Fresh Mass (g)	Center of Mass Position (mm)	Fresh Mass (g)
40	15.53 ± 0.38 a	3.51 ± 0.12 c	16.91 ± 0.44 a	3.64 ± 0.14 c	18.00 ± 0.47 a	3.82 ± 0.14 c
50	15.39 ± 0.31 b	3.99 ± 0.14 b	16.66 ± 0.32 b	4.18 ± 0.15 b	17.97 ± 0.48 a	4.28 ± 0.14 b
60	15.15 ± 0.37 c	4.18 ± 0.14 a	16.48 ± 0.36 c	4.40 ± 0.16 a	17.53 ± 0.37 b	4.91 ± 0.17 a

Note: 2.5-leaf stage, 3.0-leaf stage, and 3.5-leaf stage denote the two-leaf-and-one-emerging-leaf, three-leaf, and three-leaf-and-one-emerging-leaf stages, respectively. Data are presented as the mean ± standard deviation. Different lowercase letters within the same column indicate significant differences among root-ball moisture content treatments according to Tukey’s test (p<0.05).

**Table 2:** Experimental factors and levels.

Code	Factor		
	$X_{1}$: Root-Ball Moisture Content (%)	$X_{2}$: Seedling Age (Leaf Stage)	$X_{3}$: Seedling-Picking Frequency (s^−1^)
−1	40	2.5	4
0	50	3.0	6
1	60	3.5	8

Note: For seedling age $X_{2}$, 2.5, 3.0, and 3.5 denote the two-leaf-and-one-emerging-leaf, three-leaf, and three-leaf-and-one-emerging-leaf stages, respectively.

**Table 3:** Experimental design and results.

Run	Factor Levels			Responses			
	$X_{1}$	$X_{2}$	$X_{3}$	Reorientation Success Rate, $F_{c}$ (%)	Reorientation Height, $H_{f}$ (cm)	Successfully Ejected Seedlings, $N_{q}$	Successfully Reoriented Seedlings, $N_{c}$
1	−1	−1	0	86.67	33.6	30	26
2	1	−1	0	93.75	40.5	32	30
3	−1	1	0	83.87	38.3	31	26
4	1	1	0	84.38	41.1	32	27
5	−1	0	−1	81.25	32.5	32	26
6	1	0	−1	89.66	36.7	29	26
7	−1	0	1	84.38	36.4	32	27
8	1	0	1	83.87	42.6	31	26
9	0	−1	−1	93.1	34.7	29	27
10	0	1	−1	85.71	39.5	28	24
11	0	−1	1	87.5	40.1	32	28
12	0	1	1	81.25	40.5	32	26
13	0	0	0	85.71	39.1	28	24
14	0	0	0	86.21	38.5	29	25
15	0	0	0	86.67	39.2	30	26
16	0	0	0	87.5	40.8	32	28
17	0	0	0	85.71	39.6	28	24

**Table 4:** Analysis of variance for reorientation success rate.

Source	Sum of Squares	Degrees of Freedom	Mean Square	F Value	p Value
Model	178.04	9	19.78	14.75	0.0009
$X_{1}$	29.99	1	29.99	22.36	0.0021
$X_{2}$	83.27	1	83.27	62.07	0.0001
$X_{3}$	20.22	1	20.22	15.08	0.0060
$X_{1}X_{2}$	10.79	1	10.79	8.04	0.0252
$X_{1}X_{3}$	19.89	1	19.89	14.83	0.0063
$X_{2}X_{3}$	0.3249	1	0.3249	0.2422	0.6377
${X_{1}}^{2}$	1.76	1	1.76	1.31	0.2899
${X_{2}}^{2}$	8.90	1	8.90	6.63	0.0367
${X_{3}}^{2}$	3.59	1	3.59	2.68	0.1457
Residual	9.39	7	1.34		
Lack of Fit	7.13	3	2.38	4.20	0.0997
Pure Error	2.26	4	0.5658		
Corrected total (Cor Total)	187.43	16			

**Table 5:** Analysis of variance for reorientation height.

Source	Sum of Squares	Degrees of Freedom	Mean Square	F Value	p Value
Model	120.14	9	13.35	21.05	0.0003
$X_{1}$	50.50	1	50.50	79.63	0.0001
$X_{2}$	13.78	1	13.78	21.73	0.0023
$X_{3}$	32.80	1	32.80	51.73	0.0002
$X_{1}X_{2}$	4.20	1	4.20	6.63	0.0368
$X_{1}X_{3}$	1.00	1	1.00	1.58	0.2495
$X_{2}X_{3}$	4.84	1	4.84	7.63	0.0280
${X_{1}}^{2}$	7.76	1	7.76	12.23	0.0100
${X_{2}}^{2}$	0.3602	1	0.3602	0.5680	0.4756
${X_{3}}^{2}$	4.49	1	4.49	7.08	0.0325
Residual	4.44	7	0.6342		
Lack of Fit	1.51	3	0.5025	0.6855	0.6061
Pure Error	2.93	4	0.7330		
Corrected total (Cor Total)	124.58	16			

**Table 6:** Regression-model diagnostics.

Response	Coefficient of Variation (%)	Coefficient of Determination (*R*^2^)	Adjusted Coefficient of Determination (Adjusted *R*^2^)	Predicted Coefficient of Determination (Predicted *R*^2^)	Adequate Precision
$Y_{1}$	1.34	0.9499	0.8855	0.3727	13.2371
$Y_{2}$	2.07	0.9644	0.9185	0.7696	14.8578

## Data Availability

The raw data supporting the conclusions of this article will be made available by the authors on request.

## Appendix A

Residual diagnostics are presented in Figure A1. The normal probability plots were approximately linear, and the Shapiro–Wilk tests did not reject residual normality for either reorientation success rate (W=0.9268, p=0.1922) or reorientation height (W=0.9500, p=0.4564). The residuals-versus-predicted plots showed no evident systematic patterns, and all externally studentized residuals were within ±3. The maximum Cook’s distance was 0.497 for the reorientation-height model. For the reorientation-success model, runs 2 and 12 had Cook’s distances of 1.100 and were identified as potentially influential observations. Re-examination of the original high-speed recordings and experimental records revealed no operational, recording, or data-entry errors; therefore, both runs were retained as valid points in the planned Box–Behnken design. Nevertheless, their influence, together with the relatively low predicted $R^{2}$, indicates limited prediction stability. Accordingly, the reorientation-success model was interpreted only within the investigated experimental domain.
